# Phage treatment of multidrug-resistant bacterial infections in humans, animals, and plants: The current status and future prospects

**DOI:** 10.1016/j.imj.2025.100168

**Published:** 2025-02-05

**Authors:** Omor Faruk, Zilhas Ahmed Jewel, Sanjoy Bairagi, Mohammad Rasheduzzaman, Hindol Bagchi, Akber Subahan Mahbub Tuha, Imran Hossain, Ayon Bala, Sarafat Ali

**Affiliations:** a Department of Biotechnology and Genetic Engineering, Bangabandhu Sheikh Mujibur Rahman Science and Technology University, Gopalganj 8100, Bangladesh; b Faculty of Agriculture, Bangabandhu Sheikh Mujibur Rahman Science and Technology University, Gopalganj 8100, Bangladesh; c Department of Pathobiological Sciences, School of Veterinary Medicine, Louisiana State University, Baton Rouge, LA 70803, USA

**Keywords:** Multidrug-resistant bacteria, Phage safety, Phage-based vaccine, Biofilm disruption, Phage cocktail, Therapeutic phage

## Abstract

Phages, including the viruses that lyse bacterial pathogens, offer unique therapeutic advantages, including their capacity to lyse antibiotic-resistant bacteria and disrupt biofilms without harming the host microbiota. The lack of new effective antibiotics and the growing limitations of existing antibiotics have refocused attention on phage therapy as an option in complex clinical cases such as burn wounds, cystic fibrosis, and pneumonia. This review describes clinical cases and preclinical studies in which phage therapy has been effective in both human and veterinary medicine, and in an agricultural context. In addition, critical challenges, such as the narrow host range of bacteriophages, the possibility of bacterial resistance, and regulatory constraints on the widespread use of phage therapy, are addressed. Future directions include optimizing phage therapy through strategies ranging from phage cocktails to broadening phage host range through genetic modification, and using phages as vaccines or biocontrol agents. In the future, if phage can be efficiently delivered, maintained in a stable state, and phage–antibiotic synergy can be achieved, phage therapy will offer much needed treatment options. However, the successful implementation of phage therapy within the current standards of practice will also require the considerable development of regulatory infrastructure and greater public acceptance. In closing, this review highlights the promise of phage therapy as a critical backup or substitute for antibiotics. It proposes a new role as a significant adjunct to, or even replacement for, antibiotics in treating multidrug-resistant bacterial infections.

## Highlights

• Phage therapy targets multidrug-resistant bacterial infections.
• Offers advantages like biofilm disruption and microbiota preservation.
• Provides benefits compared to antibiotics by preserving the host's microbiota.
• Potential to serve as a replacement or complement for antibiotics.
• Faces challenges like narrow host range and regulatory hurdles.
• Future optimizations include phage cocktails and genetic modifications.

## 1 Introduction

**Fig. 1:**
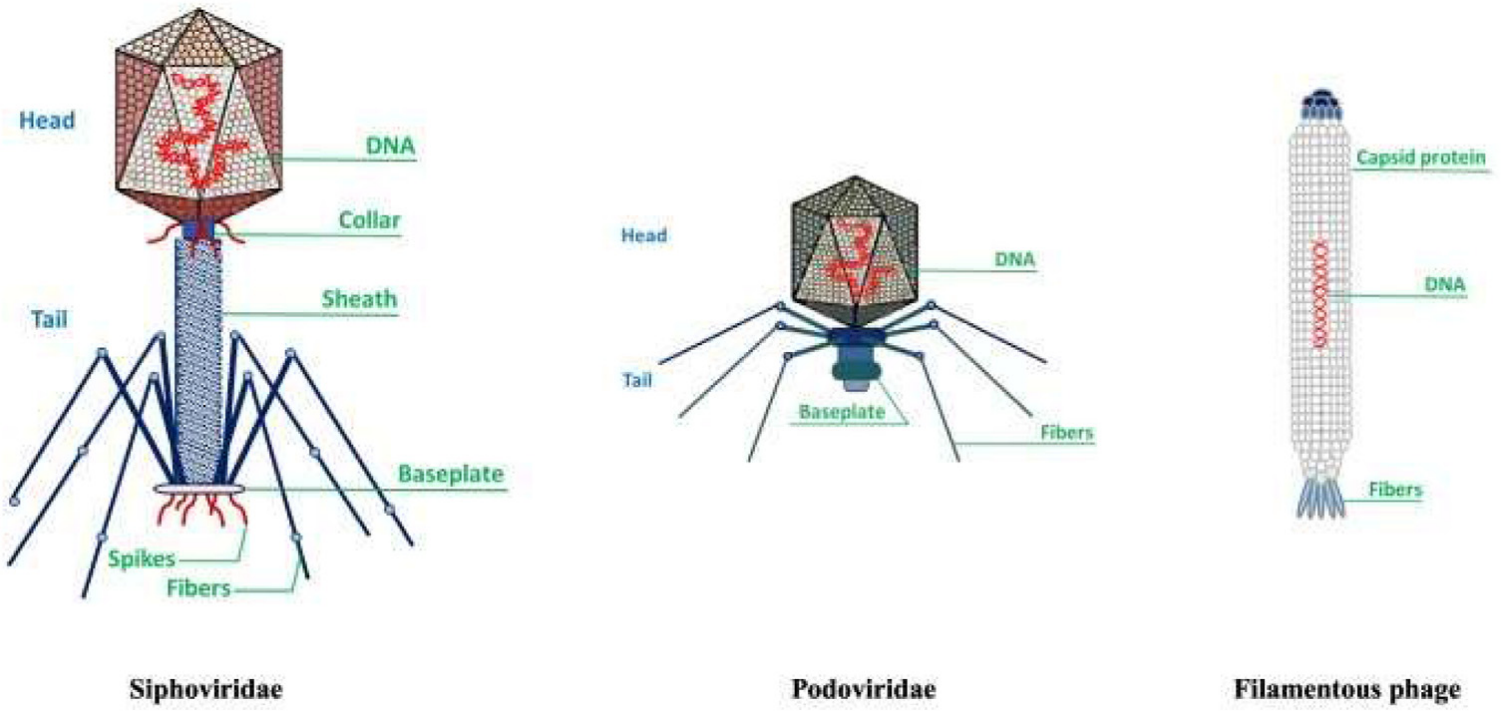
Infection by a bacteriophage: the virus's structure.^1^

**Fig. 2:**
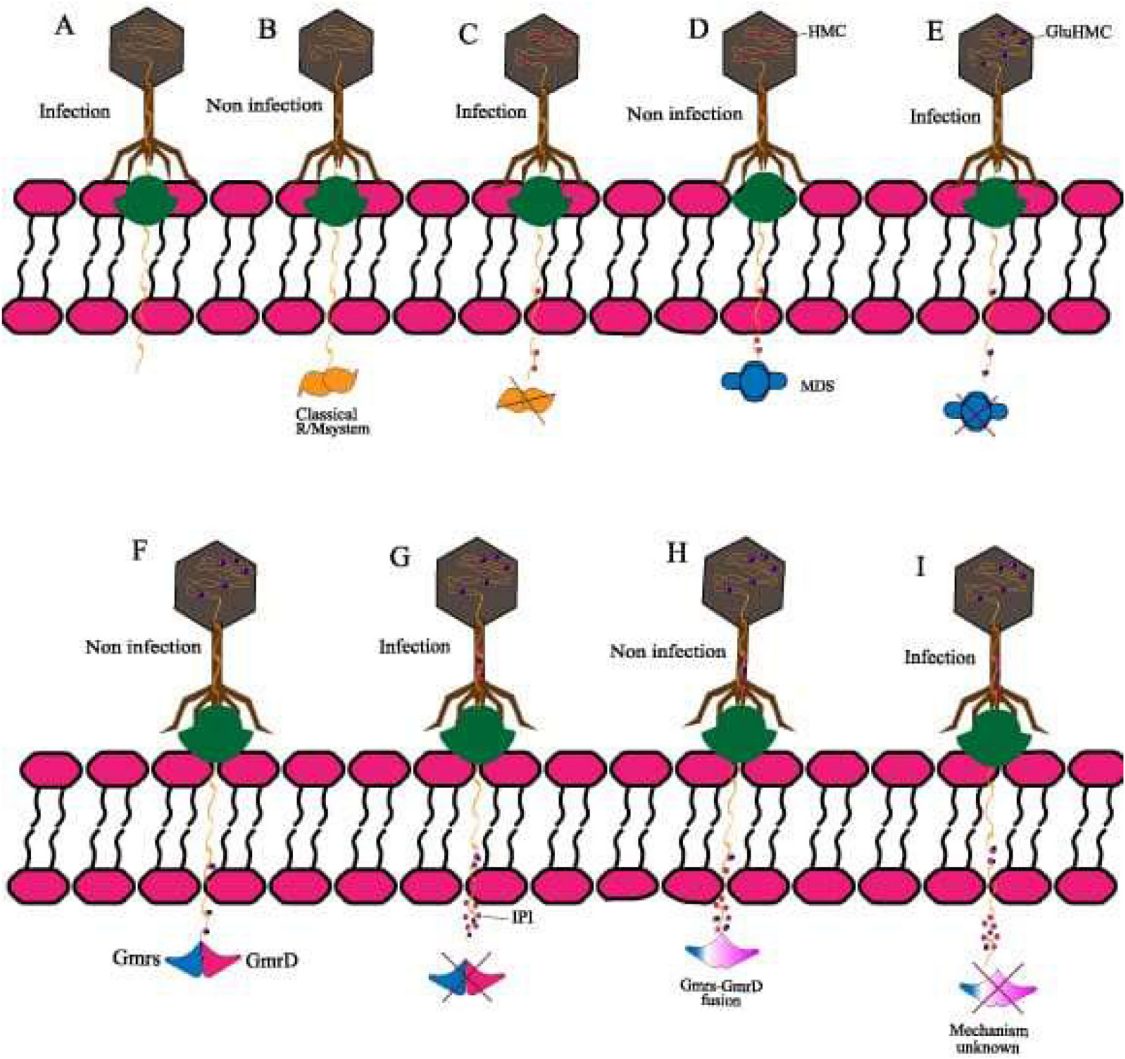
Phage T4 competes with host bacteria for survival using defense mechanisms against bacterial defense mechanisms, particularly in *Escherichia coli*. In other words, these adaptations might have included the incorporation of hydroxymethylcytosine (HMC) into the phage genome and the conversion of HMC residues or developing resistance to cleavage. In addition, phages evade bacterial defense or successful infection by expressing these phage proteins, such as interior protein I (IPI), and it is through these proteins that it may evade host restriction as well.

*Abbreviations*: HMC, hydroxymethylcytosine; IPI, interior protein I; MDS, minimal data set.

**Fig. 3:**
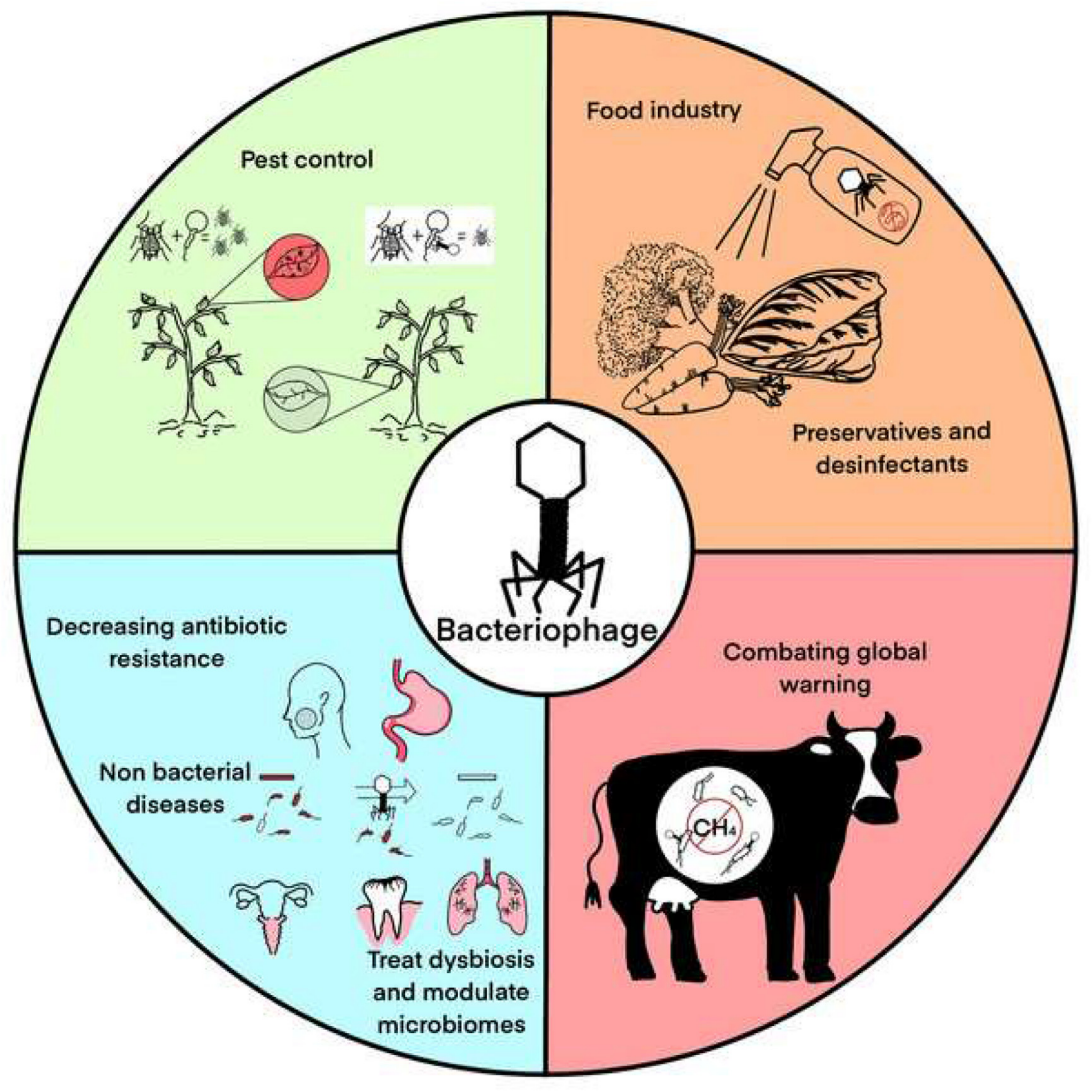
The comprehensive implementation of bacteriophage therapy within the domain of combating bacterial infections.^2^

Bacteriophages, or phages, are specialized viruses that infect bacteria for replication. As shown in Fig. 1, phages possess a protein capsid that protects their genetic material and facilitates entry into host cells.^1^ The tail structure resembles a syringe and aids attachment and injection of their genetic material. Specific phages feature complex baseplates with receptor-binding proteins for effective docking.^2^ After successful attachment, as depicted in Fig. 2 the phage injects its DNA into the bacterial cell, using the host's machinery to replicate. Eventually, new phages emerge, often destroying the host and continuing the infection cycle. Phage therapy, using bacteriophages to treat bacterial infections, was first identified by Félix d'Herelle in the early 20th century. Although promising initially, development was virtually eclipsed by the advent of antibiotics in the western world.^3^ However, Eastern Europe and the former Soviet Union continued to use phage therapy extensively, generating a rich source of empirical data.^4^ Phages operate through two primary life cycles: the lytic cycle, in which phages replicate within and then lyse bacterial cells, and the lysogenic cycle, in which phage DNA integrates into the bacterial genome and is replicated along with it, until the phage is triggered to re-enter the lytic phase. This specificity allows phages to target and eliminate bacterial pathogens without affecting human cells or the beneficial microbiota.^4^ Practical applications of phage therapy range from eradicating pests, preventing food spoilage, reducing antibiotic resistance (as illustrated in Fig. 3), treating dysbiosis, to regulating the microbiome.^5^ Phages bind to specific receptors on the bacterial surface, inject their genetic material, and hijack the bacterial machinery to produce new phage particles. This process culminates in the lysis of the bacterial cell, releasing new phages to infect other bacteria. This self-perpetuating cycle is highly effective against bacterial populations, including antibiotic-resistant microbes.^8^^,^^9^ Phage therapy has shown promise in treating multidrug-resistant (MDR) bacterial infections, particularly those associated with biofilms and intracellular pathogens. For example, phages have been used to treat severe infections where antibiotics have failed. Such cases highlight the potential of phage therapy as a complementary or alternative treatment to antibiotics. Beyond treating bacterial infections, phages are being explored for their potential in vaccine development, cancer therapy, and as gene delivery vectors. These innovative alternative uses exploit the unique properties of phages to target specific cells and deliver therapeutic agents.^8^ Despite its potential, phage therapy faces several challenges. The specificity of phages necessitates a thorough understanding of the bacterial target strains, and the development of phage resistance during treatment is a significant concern. Additionally, the regulatory landscape for phage therapy is complex, with varying requirements across different jurisdictions, complicating its clinical implementation.^4^^,^^7^ The uncertainty surrounding the patentability of naturally-occurring phages poses challenges for commercialization. Moreover, the production and manufacturing protocols for good manufacturing practice-grade phage preparations are complex and require stringent quality control measures to ensure safety and efficacy.^4^ Ongoing work is seeking to overcome these limitations using techniques such as bioinformatics and genetic engineering. For example, improving phage efficacy by genetic modification and using phage cocktails to prevent the development of resistance are promising advances. Collaborative efforts and significant funding from national and international sources are crucial for advancing phage therapy research and bridging the gap between basic research and clinical practice.^6^^,^^8^ This review aims to provide a comprehensive understanding of the current status of phage therapy, evaluating its potential for more widespread use. It also identifies and analyzes the challenges inherent in implementing effective phage treatments in medical and agricultural contexts, providing up-to-date information on this evolving field.

## 2 Objective

To contribute to an understanding of the promising role of phage therapy in the ongoing challenge presented by increasing antibiotic resistance. This work critically reviews the current state of phage therapy as an alternative treatment for MDR bacterial infections in humans, animals, and plants, with the aim of inspiring future phage therapy investigations. This review aims to:

(1) Assess the efficacy of phage therapy: we analyze therapeutic outcomes and safety profiles of phages used in human clinical settings, and veterinary and agricultural applications, highlight successful case studies, and identify potential challenges.
(2) Explore mechanisms of action: we offer insights into host range, resistance development, and the role of bacterial biofilms in the mechanisms by which bacteriophages target and eliminate MDR bacteria.^9^
(3) Identify regulatory and ethical considerations: this section discusses regulatory frameworks and ethical issues related to phage therapy in diverse settings, highlighting the need for protocol standardization and safety assessment.^10^^,^^11^
(4) Forecast future directions: we provide a forward-looking discussion of genetic approaches, personalized medicine, and the combination of phages (cocktails) to address bacterial resistance^12^ as an addition to existing treatment paradigms.

### 2.1 Lytic phage and their use in phage therapy

The lytic cycle, a crucial stage in phage growth, is critical in therapeutic applications. This cycle, also known as the “reproductive cycle” of bacteriophages, consists of six stages: attachment, invasion, transcription, biosynthesis, maturation, and lysis.^13^ These stages outline the process by which virulent phages infect and lyse the cells of their bacterial host, an essential aspect of phage therapy.^14^ Phages inject genetic material into their bacterial host by attaching to a specific receptor on the bacterial surface. The host cell replicates the phage's genetic material, providing the necessary enzymes and molecular components, thereby producing progeny phage.^15^ The lysis of the host cell occurs from the inside out, facilitated by proteins expressed by the virus, such as endolysin and holin. Small proteins called holins accumulate in the host cell's cytoplasmic membrane, reducing the degradation of peptidoglycan by the proteolytic enzyme endolysin and subsequently leading to the release of progeny phage.^16^ With their rapid and abundant reproduction, lytic phages can infect and eradicate surrounding bacteria. The rapid and abundant reproduction of lytic phages is a crucial factor in the effectiveness of phage therapy.^17^ While infecting bacterial cells, phages encounter various antiviral defenses and have developed diverse strategies to overcome these defenses.^18^ Fig. 2 illustrates the evolutionary adaptations phages employ to successfully breach the bacterial cell barrier.

### 2.2 The concept of antibiotic resistance

**Fig. 4:**
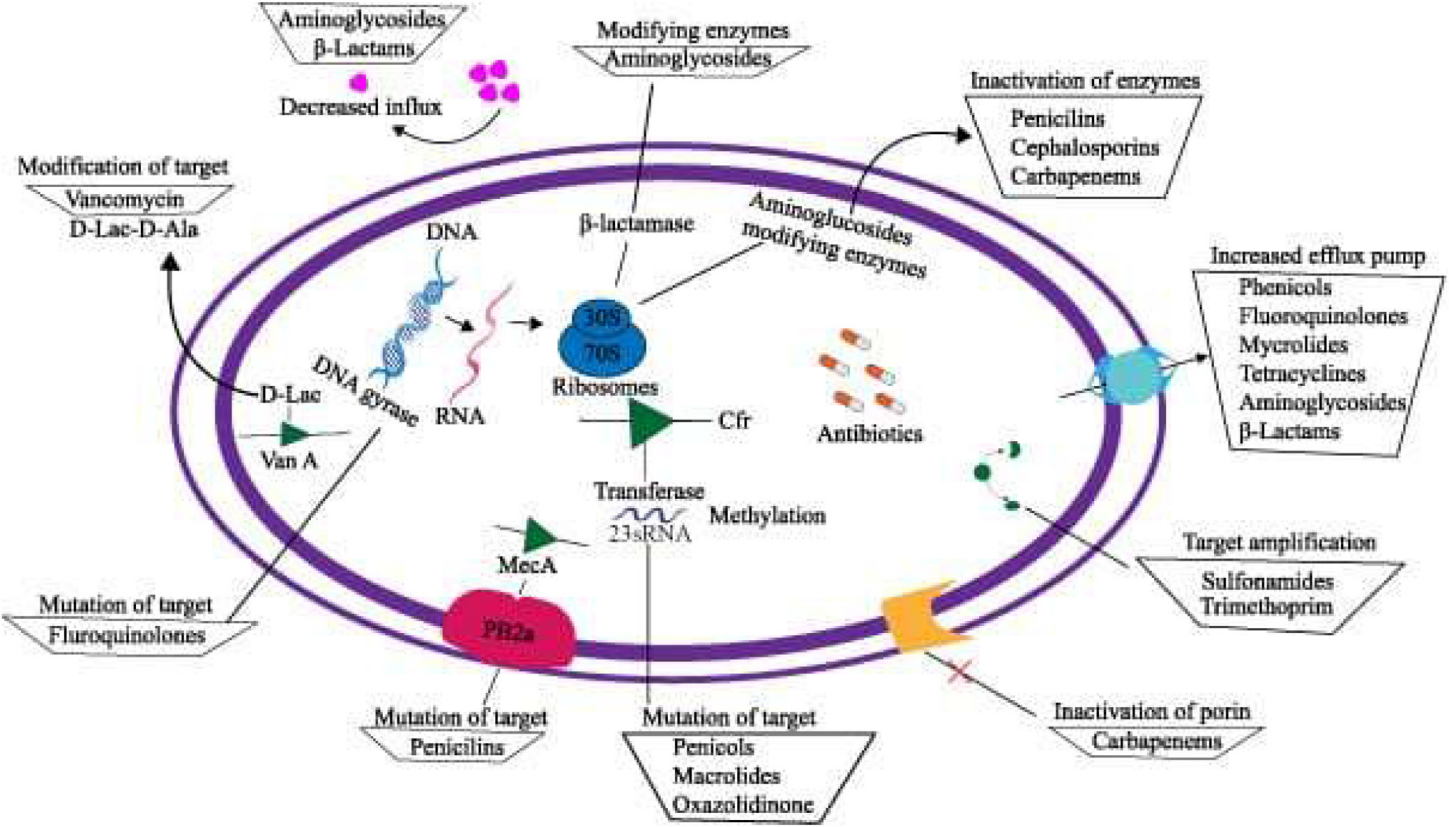
A bacterial cell's process of resistance to antibiotics is illustrated.^3^

Bacteria can develop resistance against the activity of antibiotics, rendering the drugs ineffective. The issue of antibiotic resistance has serious implications for global health.^19^ Genetic mutations and the process of horizontal gene transfer enable resistance genes to disseminate among bacteria, as illustrated in Fig. 4.^20^ The spread of antibiotic resistance is accelerated primarily by the misuse and overuse of antibiotics in human medicine and agriculture, which leads to the selection and propagation of resistant strains.^6^ The implications of antibiotic resistance are profound, affecting health, food security, and economic development, particularly in low- and middle-income countries with a high burden of infectious diseases.^21^ Fig. 4 shows that bacteria accumulate resistance either through mutations or, in the case of plasmids, by taking up resistance genes from other bacteria through horizontal gene transfer.^22^ Plasmids have the potential to transfer multiple resistance gene cassettes, thereby further amplifying the spread of resistance among bacterial populations.^23^ In addition, antibiotic residues in the environment, resulting from improper disposal, can exacerbate resistance levels. Increased mortality rates, higher medical costs, and decreased efficacy of current antimicrobial agents, all contribute to the mounting public health threat presented by antibiotic resistance, which warrants immediate intervention.^24^

### 2.3 Phage therapy *versus* antibiotic therapy

The targeted action and effectiveness against antibiotic-resistant bacteria make phage therapy an increasingly attractive alternative to antibiotics. Unlike antibiotics, which indiscriminately kill both pathogenic and beneficial bacteria, phages kill only pathogenic bacteria causing minimal disruption to the host's normal microbiota.^25^ This specificity also decreases the incidence of side effects because phages do not kill non-target cells.^26^ Moreover, phage therapy can potentially degrade biofilms that may affect beneficial microbiota.^4^^,^^27^ However, because of its specificity, phage minimizes side effects compared with broad-spectrum antibiotics. Using bacteriophages (*i.e.*, viruses that infect bacteria) is a potential means to fight drug-resistant infections as they can evolve alongside the bacteria they infect, remaining effective against resistant strains.^28^^,^^29^ Phages are also effective at degrading biofilms, which are often resistant to antibiotics. Phage therapy is associated with low inherent toxicity and low dose requirements.^30^ Its application in clinical trials has demonstrated promising results, mainly when combined with antibiotics.^28^ Phage therapy, however, has some limitations, for example, the host specificity of phages and the resulting need for personalized treatment, and the immune response to phages.^31^ However, despite these hurdles, the ability of phage therapy to treat antibiotic resistance and biofilm-related infections, which traditional antibiotics cannot, makes it a promising alternative.^32^

### 2.4 Phages as industrial medicinal products: the regulatory framework

**Fig. 5:**
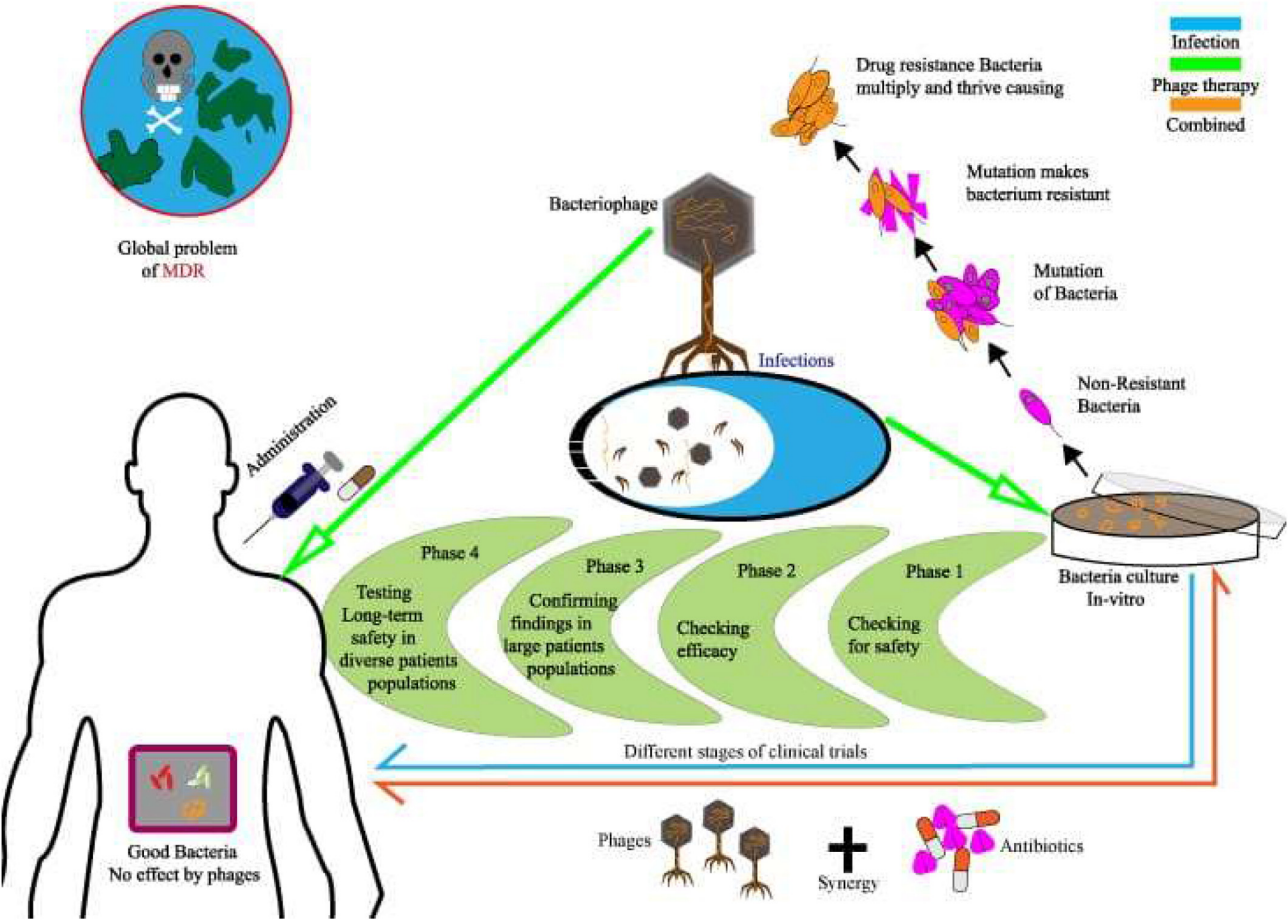
Phage therapeutics: from concepts to applications and future directions.^3^

*Note*: here, drug resistance is caused by bacteria. Infections can occur in bones and joints, the urinary tract, biofilm, the heart and lungs, the gastrointestinal bloodstream, and skin and soft tissue. *Abbreviation*: MDR, multi drug resistance.

The escalating prevalence of MDR bacteria presents a formidable global health challenge, necessitating urgent interventions, including exploring novel antibacterials. Notably, phages operate within a regulatory framework distinct from traditional antibiotics.^33^ Since 2011, phages have been classified as drugs in the USA. By contrast, the European Medicines Agency (EMA) defines medicinal products within the European Union as substances or compound formulations intended for therapeutic, prophylactic, or diagnostic purposes or the improvement, correction, or modulation of physiological functions through pharmacological, immunological, or metabolic modalities.^34^^,^^35^ Phage therapy offers unique attributes compared with antibiotics, notably its specificity toward particular bacterial hosts and its minimal impact on the commensal microflora.^36^^,^^37^ Unlike conventional antibiotics, phages exert their antimicrobial effects in a targeted manner, thereby circumventing broader disruption of microbial ecosystems. These distinct features underscore the potential of phage therapy as a viable option or adjunct to established antibiotic regimens in combating MDR bacterial infections, heralding a new era in infectious disease management that is reflected in Fig. 5.^38^^,^^39^

### 2.5 Overview of the methodology for phage therapy

**Fig. 6:**
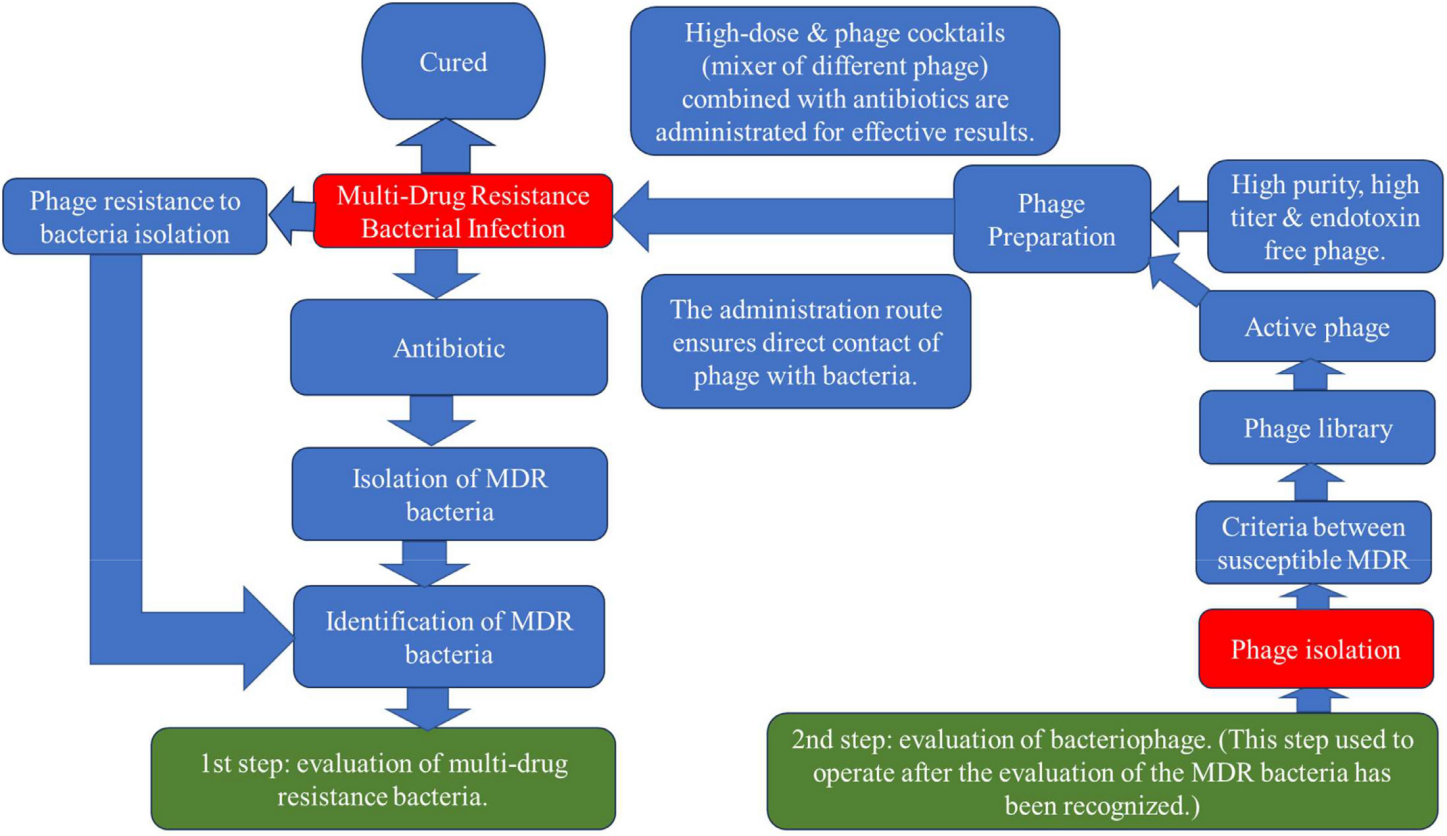
An oversimplified view of our workflow process.

*Abbreviation*: MDR, multi-drug resistance.

Before initiating phage treatment, the infecting bacteria must first be successfully grown, then identified using nonculture-based techniques. Finally, the isolate is tested for its responsiveness to phages. Phage therapy is often reserved for situations where antibiotics are no longer effective or when the infecting bacteria are resistant to numerous drugs.^40^ Successful phage treatment depends on isolating and identifying the infectious pathogens responsible for the disease. Following the discovery of a bacterial infection in a clinical environment, bacteria are isolated and identified, as shown in Fig. 6.^41^ As a result of advances in diagnostic procedures, clinical bacteriology increasingly employs nonculture-based methods such as PCR, gene sequencing, matrix-assisted laser desorption/ionization time-of-flight mass spectrometry (MALDI-TOF MS), and immunological assays.^42^ Phage treatment is not appropriate for use in nonbacterial infections such as those caused by viruses, fungi, or parasites.^43^

### 2.6 Route of administration for phage preparations

The optimal administration of phage preparations should ensure sufficient phage–bacteria interactions, while avoiding procedures that could inactivate phages. Protecting phages from stomach acid is vital for treating gastrointestinal infections.^44^ Antiseptics and other therapies that might deactivate phages should be avoided. Based on their nanoparticle-like characteristics, phages show limited diffusion within the body, meaning that close contact with bacterial pathogens is essential.^45^^,^^46^ Topical phage-infused lotions or powders may effectively treat skin infections.^47^ aerosolized preparations are recommended for respiratory infections, and infusion preparations may be suitable for bloodstream infections.^48^ Phage-containing capsules designed to withstand stomach acid are preferred for gastrointestinal illnesses, though their effectiveness is still being evaluated.^44^

### 2.7 Strategy for phage preparation administration

Phage isolation and selection co-occur in phage therapy.^49^ Two primary models for phage selection exist: the first involves targeting a broader range of bacterial strains with phage mixtures (such as intestiphage and prophage), potentially reducing resistance (Fig. 6). These mixtures are reportedly more effective than individual phage, paving the way for commercial therapeutic phage formulations.^50^ The second method isolates pathogenic bacteria from patients and compares them with a well-characterized phage collection, yielding better clinical outcomes than using generic phage in some cases.^51^ (Fig. 6). This approach, however, may also promote the spread of antibiotic-resistant bacteria.^52^^,^^53^

### 2.8 Phage detection and manufacture of a stock solution

Effective antibacterial treatment relies on a diverse library of bacteriophages, which can be specialized or general in their host range.^54^^,^^55^ It is essential to ensure that the bacteria targeted are phage-sensitive, particularly regarding MDR strains such as *Pseudomonas aeruginosa* and *Staphylococcus aureus*.^56^ The phage collection should include lytic and virulent phages, with stringent enrolment criteria to ensure safety and the absence of undesirable genes.^54^^,^^57^ Phage isolation begins with enrichment from raw sewage, followed by centrifugation and filtration to remove contaminants. The process involves mixing sewage and lysogeny broth (LB) broth with a bacterial strain and incubating overnight. Enrichments are filtered again, and further enrichment is conducted to increase phage yield. Plaques observed on bacterial lawns are purified through multiple rounds to isolate uniform phages.^58^ Phage cocktails can enhance efficacy against bacterial strains and reduce resistance.^48^ The methodology includes mixing host strains with diluted phage stock on agar plates, incubating overnight, and filtering for clarity, as depicted in Fig. 6.^58^

### 2.9 Evaluating the efficacy of phage therapeutic interventions

The development of phage-resistant strains should be monitored frequently during phage therapy. It is essential to monitor the patient's condition regularly and to analyze clinical samples from bacterial infection sites quickly to determine the efficacy of treatment, phage resistance, and the optimal phage concentration. Phages should be replaced as soon as specific phage-resistant bacteria arise. When bacterial strains resistant to library phages develop phage resistance, using these strains as hosts makes it easier to screen various materials for new phages a.^59^ If such novel phages fit the criteria, they can be added to the library continuously and prepared following the approach described above. Theoretically, phage therapy is an example of personalized medicine applied to treat bacterial infections.^48^ Additionally, phage-resistant strains might sometimes become less virulent.^60^^,^^61^ Changes in phage resistance can affect antibiotic susceptibility.^62^ Therefore, concurrently evaluating antibiotic resistance profiles in phage-resistant organisms is essential. Furthermore, examining how phages and antibiotics synergize to prevent bacterial infection in clinical settings may help guide treatment plans. These concepts, depicted in Figs. 5 and 6.^63^ support phage monotherapy or phage therapy in conjunction with antibiotics.

### 2.10 MDR infections in humans targeted by phage therapy

#### 2.10.1 Burn infections

*P. aeruginosa* is a significant cause of severe infections in burn patients with a compromised skin barrier and immune system.^64^ Its rapid colonization can lead to severe complications such as bacteremia and septic shock, with infections being a leading cause of burn-related deaths.^65^^,^^66^ The resistance of this bacterium to a number of antibiotics complicates treatment, and approximately half of all burn-related fatalities are linked to infections, particularly by *P. aeruginosa*. Phage therapy has been explored as an alternative to antibiotic therapy in this context.^67^ In one study^68^ burn-injured mice infected with *P. aeruginosa* were treated with a phage cocktail via different administration routes: intramuscular (i.m.), subcutaneous (s.c.), and intraperitoneal (i.p.). The i.p. route offered the best protection (87% survival), significantly improving mortality rates compared with no treatment. Phage administration reduced mortality by 72% with i.m. administration and 78% with s.c. administration, while i.p. administration provided the highest relative benefit. Overall, phage therapy via the i.p. route enhanced survival in injured, infected mice.^69^

#### 2.10.2 Cystic fibrosis

Cystic fibrosis (CF) is a hereditary condition caused by mutations in the cystic fibrosis transmembrane regulator (CFTR) gene, particularly the p. Phe508del mutation detected in over 70% of cases.^70^^,^^71^ This mutation leads to impaired chloride ion transport, resulting in mucus buildup in the lungs, compromised mucociliary clearance, and increased susceptibility to infections. *P. aeruginosa* is the predominant pathogen responsible for infection in CF patients, with approximately 17.9% of strains being MDR.^72^ In addition to *P. aeruginosa*, other pathogens such as *Mycobacterium abscessus* are becoming more prevalent in CF patients. Based on their resistance patterns, infecting bacteria often require a combination of at least three antimicrobial agents, as reflected in Table 1A.^73^ Phage therapy has produced promising results in CF patients. For example, a 15-year-old CF patient with widespread *M. abscessus* infection showed significant lung function improvement after receiving phage therapy, which involved applying genetically engineered phages directly to infected areas and administering them intravenously (i.v.) over 32 weeks.^74^ Similarly, a study involving a 26-year-old CF patient treated for MDR *P. aeruginosa* demonstrated successful ambulation and removal from the lung transplant waitlist following eight weeks of phage therapy, as detailed in Table 1A.^75^^,^^76^ These cases highlight the potential of phage therapy in managing bacterial infections in CF patients, underlining the need for further research into its safety and effectiveness.

**Table 1A:** Phage therapy efficacy rate on cystic fibrosis.^1^, ^2^, ^3^, ^4^

Organism	Demographics	Infectious syndrome	Route of phage administration	No. of phage administration and duration	Highest dose of phage administered (PFU)	Result
The Liverpool epidemic strain as LES431 of *P. aeruginosa*	26-year-old female	Cystic fibrosis exacerbation	Intravenous (i.v.)	4 phages; 8 weeks	4 × 10^9^	Clinical improvement 7 days into therapy; no CF exacerbations within 100 days following end of phage therapy.
*Achromobacter* species	10-year-old female	Cystic fibrosis exacerbation in a cystic fibrosis patient	Intravenous (i.v.)	1 phage; 28 days (14 days without antibiotics and 14 days with antibiotics)	Not described	FEV_1_ improved when administered with antibiotics; *Achromobacter* sp. could not be recovered up to 16 weeks after treatment.
*Achromobacter xylosoxidans*	17-year-old female	Cystic fibrosis exacerbation in a cystic fibrosis patient	Oral and nebulized	2 phages, 20 days	3 × 10^8^	Dyspnea resolved and cough decreased; FEV_1_ increased from 54% to 84%.

*Abbreviations*: PFU, plaque-forming units**;** LES, Liverpool epidemic strain; CF, cystic fibrosis; FEV_1_, forced expiratory volume in 1 second.

#### 2.10.3 Pneumonia

For the therapeutic application of bacteriophages to treat antibiotic-resistant infections, the interaction between phages and bacteria within the living organism must be considered.^77^ A mathematical model was included in the integrative paradigm suggested by Delattre et al., which combined *in vivo* studies with *in vitro* laboratory investigations. This technology examined the interaction between bacteria and bacteriophages in the case of pneumonia caused by a highly pathogenic *Escherichia coli* strain (Table 1B). The model made it easier to estimate a number of critical aspects related to the effectiveness of phage therapy, such as the effect of phage dosage and delivery route, the synergistic effect of phages, and the innate immune response mounted to eliminate the pathogen.

**Table 1B:** Analytical prospects of phage therapy in rats on pneumonia.^5^

Uninfected and treated	Infected and untreated	Infected and treated
Intratracheal (i.t.) (8 log_10_ PFU; *n* = 16)	Intratracheal (i.t.) (7.6 log_10_ CFU; *n* = 45)	Intratracheal (i.t.) Hi (8.6 log_10_ PFU; *n* = 10)
Intravenous (i.v.) (8 log_10_ PFU; *n* = 37)		Intratracheal (i.t.) Med (7.6 log_10_ PFU; *n* = 25)
		Intratracheal (i.t.) Lo (5.6 log_10_ PFU; *n* = 24)
		Intravenous (i.v.) Med (7.6 log_10_ CFU; *n* = 16
		Intravenous (i.v.) Lo (8 log_10_ CFU; *n* = 50)

*Abbreviations*: PFU, plaque-forming units; Hi, high; Med, median; Lo, low; CFU, colony-forming units.

### 2.11 Phage distribution in non-infected murine models

The study by Delattre et al. evaluated two methods of phage delivery, namely intratracheal (i.t.) and intravenous (i.v.), in non-infected mice. The mice were administered 8 log_10_ plaque-forming units (PFUs) of phage 536_P1 as a single dose. Four hours after phage administration, the peak median phage concentration in the lungs was 5.1 [4.7, 5.5] log_10_ PFU/g for i.v. injection compared with a higher value of 7.2 [6.8, 7.8] log_10_ PFU/g for i.t. administration. After i.v. administration, only a small portion, approximately 0.02%, of the phage reached the lung compartment. This resulted in a significant difference of nearly 2 logs_10_ compared with the results with i.t. administration, with exposure to the lung compartment being approximately 5 000 times lower. The absorption rate into the lung compartment, known as the Ka, was estimated to be 0.3 h^˗1^. The rate at which the i.t.-administered phage was eliminated from the lungs was slow, with a half-life of 12 h (log_2_/Ka [IT]), whereas the i.v.-administered phage was eliminated considerably faster, with an estimated half-life of 3 h.

### 2.12 Bacterial dynamics in untreated infected mice

Using emitted luminescence and colony-forming unit equivalents (CFUeq/g), Delattre et al. (2022a) demonstrated significant variation in lung bacterial burden among 45 untreated mice after i.t. injection of *E. coli* strain 536. By allowing for the assessment of phage efficacy over a range of clinically meaningful values, the range of bacterial loads at 2 h post-infection was 5.0 log_10_ CFUeq/g to 9.9 log_10_ CFUeq/g. Various efficacious inoculums also provided information regarding the bacterial density threshold, beyond which the host's immune system cannot contain the infection. If the bacterial burden at the 2-hour post-infection evaluation was more than 6.8 log_10_ CFUeq/g, all of the animals died within 72 h. The continual increase in bacterial density was responsible for the observed mortality. Conversely, bacterial densities were often shown to decrease below this threshold, probably owing to the immune response eliminating the infection.

### 2.13 Bacterial dynamics in rodents left untreated after infection

The analytical data of phage therapy in rat models of pneumonia are presented in Table 1B. Rodents were infected using a similar methodology as previously described for mice (*n* = 138), and two hours after infection, they were administered different doses of phage 536_P1 (low: 5.6 log_10_ PFU, medium: 6.6 log_10_ PFU, high: 7.6 log_10_ PFU) via the i.v. or i.t. route. Compared with that in the lungs of their untreated counterparts, the abundance of phage in the lungs of the treated rats increased significantly by 2- to 3-log_10_. This implied effective interactions with bacteria and rapid phage growth. At 4 h after phage delivery (6 h after bacterial infection), the median phage titer among uninfected animals undergoing i.t. therapy was 7.1 [7.1, 7.4] log_10_ PFU/g. By contrast, 59 mice who received i.t. treatment after infection showed a median phage titer of 9.6 [9.3, 9.9] log_10_ PFU/g. The phage concentration in i.v.-treated animals was 8.4 (7.0, 9.1) log_10_ PFU/g, whereas the phage concentration in non-infected animals receiving the same therapy was 5.1 [4.7, 5.5] log_10_ PFU/g.

### 2.14 Implementation of phage therapy in plants

The effective control of bacterial plant diseases by antibiotics and bactericidal agents has been vitally important over the years; however, the adverse effects on the environment and human health have become apparent. In addition, the high occurrence of bacteria resistant to antibiotics and pesticides, such as *Erwinia amylovora, Pseudomonas syringae, Xanthomonas campestris, Xanthomonas citri*, and *Xanthomonas citrumelonis*, coupled with the slow progress in developing new antibiotics that are effective against these bacteria, has redirected the research focus toward exploring alternative biocontrol agents for managing bacterial plant diseases.^78^ The efficacy of phage therapy has therefore been tested on several plants, as detailed in Table 2. The need for organic, antibiotic-free products among consumers is credited with the resurgence of phage therapy in treating plant diseases.^79^ There are several methods by which phage can be applied to plants, with soil-based delivery appearing to be promising with respect to enhancing phage persistence and efficacy. This method achieves systemic disease control as the phages translocate from the roots to the leaves, specifically targeting pathogenic bacteria and leaving beneficial bacteria unaffected. Phage therapy is also a promising alternative to traditional chemical pesticides.^80^ As a result of their adaptability to bacterial resistance and environmentally-safe nature, phages are beneficial for sustainable agriculture. However, many limitations exist; for example, phages are environmentally sensitive and may be destabilized or compromised in terms of efficacy under field conditions because of factors such as UV light, temperature, and pH.^81^ Although beneficial for precision, their narrow host range presents a bottleneck in ecosystems containing a heterogeneous range of pathogenic strains.^82^ Fortunately, the risk of bacteria becoming resistant through mutations at their receptors or CRISPR defenses in bacteria is typically less than with antibiotics. The widespread adoption of phage therapy is further inhibited by regulatory challenges associated with testing the safety and efficacy of bacteria-killing phages.^83^ These problems are the subject of contemporary innovations, such as engineering phages with broader host ranges, stabilizing phages through encapsulation into nanocarriers, and introducing phage-derived enzymes as direct antimicrobials.^84^ Phage therapy presents challenges but is also a promising sustainable method to manage plant bacterial infections, which may prove valuable for specific agricultural applications.^85^

**Table 2:** Phage therapy's effectiveness rate in plant systems.^6^, ^7^, ^8^, ^9^, ^10^, ^11^, ^12^

Target diseases and pathogens	Bacteriophages	Hosts	Treatment methods	Test conditions	Control efficacy (reduction of disease incidence, %)
Bacterial wilt: *Ralstonia solanacearum*	Single (RsPod1EGY)	Tomato	Soil drench	Greenhouse	100.0
	Cocktail (NJ-P3, NB-P21, NC-P34, NN-P42)	Tomato	Soil drench	Greenhouse	80.0
				Field	
	Cocktail (M5, M8)	Banana	Soil treatment	Greenhouse	100.0
Bacterial blight: *Xanthomonas oryzae pv. oryzae*	Cocktail (P4L, P43M, P23M1)	Rice	Spray	Field	50.8
Black rot: *X. campestris pv. campestris*	Single (XcpSFC211)	Broccoli	Spray	Greenhouse	60.0
				Field	16.7–55.0
Bacterial spot: *X. euvesicatoria*	Single (KФ1)	Pepper	Spray	Greenhouse	50.0–67.0

### 2.15 Exploring the integration of phage therapy in the care of livestock and companion animals

The re-emergence of MDR bacteria has increased interest in phage therapy as an alternative to antibiotics in livestock and companion animals.^86^ The ability of phage therapy to control pathogenic bacteria in animals is promising.^87^ Furthermore, the application of phage therapy in animals has gained more social acceptance than that in humans; however, international cooperation to overcome logistical and regulatory hurdles is still needed.^88^ In one study, a lyophilized bacteriophage cocktail effectively controlled MDR *Salmonella* in broiler chickens, with no accompanying clinical signs or mortality (0%). In particular, the tissue architecture of the liver and cecum was normal following phage treatment, whereas the positive control showed significant pathological changes.^89^ Furthermore, performance improved, growth rates increased, and weight gain and feed intake were higher in the phage-treated group than in the control group. Finally, the study found that lyophilization improves phage stability for long-term storage.^90^ In another study, the isolated lytic bacteriophage SD04 was shown to effectively infect *Aeromonas schubertii*, a severe pathogen of *Channa maculata*.^91^ Phage SD04 was shown to have high stability after being stored at 4°C for 12 months, a large average burst size, a broad host range, and high specificity. Following phage therapy, the survival rate of *C. maculata* was high, suggesting that phage therapy is a potentially safe and effective alternative to antibiotics for preventing *A. schubertii* infection. Phage SD04 is a promising therapeutic option for aquaculture. In addition, initial findings with phage AhFM11 showed lytic activity against hypervirulent *Aeromonas hydrophila* strains, thereby indicating considerable therapeutic potential.^92^ Phage AhFM11 harbors no known virulence or antibiotic resistance genes, improving its safety for use in aquaculture and food.^93^ *In vivo* studies indicated over 93% protection against virulent *A. hydrophila* strains, and its effectiveness was also demonstrated *in vitro* food decontamination studies. These results suggest that phage AhFM11 may be used as a non-antibiotic alternative to treat *A. hydrophila* infections and decrease the bacterial load in food products.^94^

### 2.16 The drawbacks of phage therapy

Phage therapy presents promising applications in public health, agriculture, veterinary science, and food safety but has notable drawbacks. Key disadvantages include its limited host range, selectivity, and potential cytotoxic and immunogenic reactions. Notably, phage therapy is ineffective against intracellular bacteria such as *Salmonella* because phages cannot penetrate eukaryotic cells.^95^ Furthermore, specific phages may also mediate gene transfer to bacteria, transferring bacterial toxins or increasing pathogenicity, thus raising concerns about the spread of virulence factors or antibiotic resistance.^96^^,^^97^ In some cases, phage treatment could result in rapid bacterial lysis and the release of endotoxins, which could trigger major inflammatory responses.^98^^,^^99^ In addition, resistance mechanisms have evolved among bacteria, for example, by modifying receptors to avoid phage adsorption.^98^^,^^100^ The viability and effectiveness of phage therapy are affected by environmental factors, such as pH and temperature.^6^ Another challenge facing phage is the host immune response; the human body can recognize and neutralize phages, which may adversely affect their therapeutic potential. Finally, regulatory barriers are significant and require a unique phage characterization, production, and clinical testing framework to establish the safety and efficacy of phage therapy.^101^ Ensuring phage viability and escaping immune surveillance are fundamental challenges that must be overcome prior to the application of phage-based treatments. Furthermore, repeated administration can lead to patients developing anti-phage antibodies that further complicate treatment.^102^ The effectiveness of phage therapy is complicated by the rapid emergence of phage-resistant strains.^8^ Resistance development in bacteria is primarily driven by mutations, which are usually accompanied by a fitness cost (*e.g.*, reduced virulence or impaired biofilm formation).^103^ Furthermore, the asymmetry between bacteria and phages limits coevolution because phages cannot evolve sufficiently rapidly to respond to resistant bacterial strains.^8^ However, the diversity and narrow spectrum of activity of phages proves another challenge in gaining approval for phages as therapeutic drugs.^104^^,^^105^ Antibiotic synergy testing is not routinely conducted in the pipeline, and for phage therapy, producing a long-term stable biologic has proved difficult. Translating *in vitro* findings into clinical settings is complicated by the absence of randomized controlled trials.^106^ Additionally, characterizing suitable phages for therapy is a time-consuming procedure, and the broad host range of phages is a significant obstacle.^107^ Regulatory agencies also lack standardized guidelines for phage therapeutic use, and phage therapy is not typically covered by public health insurance, raising the costs of healthcare.^26^ Previous trials failing to show expected results because of one-size-fits-all products highlight the challenge of conducting high-quality randomized controlled trials for bacteriophage therapy. Complying with good manufacturing practices for personalized bacteriophage therapy can be complex and expensive, hindering timely treatment.^108^ Additionally, the development of bacteriophage resistance during therapy remains a significant challenge for the efficacy of bacteriophage therapy.^8^ Treatment outcomes vary as all of these evaluations are subjective and based on standardized tests.^11^ Studies of bacteriophages, particularly in the gut ecosystem, are complicated by the lack of a universal marker gene for comprehensive identification and, in many cases, low sequence homology with classified phages.^109^ Culturing specific gut bacteria and creating environmental conditions within the gut required for phage replication is complex, making isolating novel gut phages difficult.^110^ More research is needed to determine the stimuli that activate prophage induction.^109^ Understanding phage–host interactions and the genetic basis for resistance may lead to the design of effective phage cocktails and strategies to prevent resistance.^111^ Elucidating the complex phage resistance mechanisms that *P. aeruginosa* has evolved is crucial to creating effective therapeutic strategies. Moreover, it is evident that phage resistance phenotypic trade-offs are inversely related to antibiotic resistance and biofilm formation.

### 2.17 Overcoming the limitations of phage-based therapeutic approaches

Phage cocktails, which combine several phage types to gain greater host breadth than any individual phage, are being developed to improve phage therapy and could be used for preemptive treatment and to limit phage-resistant bacterial strains.^112^^,^^113^ Genetic engineering has also been applied to decrease phage cytotoxicity and immunogenicity.^114^ Researchers are exploring phages that do not cause lysis, using essential proteins such as endolysins to promote bacterial cell lysis while avoiding the release of harmful progeny. Phage proteins can mitigate the risks associated with live phage, such as horizontal gene transfer.^115^ The innovative bacteriophage recombineering of electroporated DNA (BRED) technique allows for genetic alterations in bacterial genomes using lytic and temperate phages.^116^ While chemical-based transformation methods have been explored, electroporation remains the standard for phage genomic engineering.^117^^,^^118^ Genetic engineering has also targeted the phage host range, as demonstrated by modifications to the T7 phage to produce an endo sialidase, which enhances infection rates in K1 *E. coli* strains.^119^^,^^120^ Research on genetically modified temperate phages has focused on creating stable lytic states, providing a more accessible option than that offered by lytic phage alone.^121^ Future studies should investigate strategies to evade the human immune response to further increase the effectiveness of phage therapy.^111^^,^^122^ Furthermore, standardized regulatory frameworks are needed to characterize phage and test their clinical use, so that acceptance can be gained from regulatory agencies.^123^ Further research is also needed on the practical applications of phage in vaccine development, cancer immunotherapy, and gene delivery systems. Clinical trials should validate the efficacy of phage therapy in clinical practice to treat infections, especially antibiotic-resistant infections.^124^ Social acceptance of phage therapy is also essential to the future development of phage therapy.^11^ A standardized database of phage information available globally is crucial for the efficient and standardized use of phage.^125^^,^^126^ For therapy to advance, phage resistance and immune recognition must be addressed.^127^ Future studies conducting high-quality randomized controlled trials should improve the prediction of bacteriophage therapy outcomes.^108^ Bacteriophage–antibiotic interactions should also be investigated.^128^ Furthermore, an understanding of phage immune neutralization mechanisms is needed.^129^ The effects of phage on human health (especially the gut microbiota) should be researched in-depth.^110^ Triggers of prophage induction should be identified. Phage vaccines should also be tested *in vivo* for diseases such as inflammatory bowel disease, obesity, and type 2 diabetes. The effect of phage on the modulation of gut metabolites warrants further study.^130^ Accordingly, understanding phage–bacteria coevolution mechanisms is vital.^131^ Finally, data regarding phage resistance and susceptibility to antibiotics in a *mexB* mutant may aid our understanding of the MexAB OprM complex. Advanced methods may be used to discover other resistance genes.^132^ and investigating intermediate-resistant mutants might reveal new resistance pathways.^133^ The future of phage therapy involves overcoming current barriers by engineering phages with improved intracellular targeting and combining phages with antibiotics to prevent resistance.^45^^,^^135^ Nanoparticle encapsulation is a method for developing formulations of intracellular bacteria with improved delivery.^135^ This ongoing research aimed at overcoming deficiencies in current phage therapies and optimizing the phage response to MDR bacteria, has significant potential benefits for the treatment of urgent healthcare issues and the improvement of patient outcomes.^136^

## 3 Discussion

Therapeutic phages must be assessed for *in vivo* efficacy and this requires highly pure preparations free of contaminants such as endotoxins and bacterial debris.^137^ Methods such as endotoxin removal columns and microfiltration can help purify phage preparations.^138^ Rahmani et al.^138^ demonstrated the effectiveness of phage TPR7 against an MDR *E. coli* strain in a mouse model, showing equal efficacy to gentamicin. Chhibber et al.^139^ found that inflammation and bacterial burden in diabetic mice was significantly reduced by phage MR-10 compared with that in controls, with results comparable to the antibiotic linezolid. These studies highlight the potential of phage therapy against MDR bacteria.^140^ In plant pathology, new phages, such as RSSKD1 and RSSKD2, have been identified to combat *Ralstonia solanacearum*, while Rombouts et al.^141^ tested a phage cocktail against *P. syringae*, and further research may enhance efficacy. Lee et al.^142^ explored the use of phage endolysin, which effectively reduced bacterial concentrations, and Wittmann et al.^143^ reported that transgenic tomato plants expressing endolysins showed reduced disease symptomatology, indicating its potential in plant defense.^144^ Phage therapy is becoming an attractive alternative to combat MDR bacteria and to meet a critical need in modern medicine in the face of increasing antibiotic-resistant infections.^4^ The use of bacteriophages (*i.e.*, viruses that specifically infect bacteria) to treat bacterial infections has existed throughout most of the 20th century^8^^,^^145^ but their use was superseded with the advent of antibiotics. The potential of phage therapy has been rediscovered in recent times and shows promise against several highly-resistant pathogens, such as *P. aeruginosa* and *S. aureus*, as well as in combination with other antibiotics to increase their antibacterial effects, as demonstrated by phage endolysins and colistin against *Acinetobacter baumannii*.^104^ Phage therapy is now being explored as a practical option for targeting drug-resistant bacteria, disrupting biofilms, and reaching intracellular pathogens, which are limitations of current antibiotics.^146^ Phages in consort with antibiotics can also induce phage–antibiotic synergy, in which phages have been shown to reduce phage-resistant mutants and increase antibiotic efficacy.^147^ However, limitations such as phage stability and immune response regulatory hurdles^148^ must be overcome before phage therapy can be applied in mainstream medical practice. Future work will include improving phage delivery mechanisms, engineering phages to accurately target intracellular bacteria, and devising regulatory schemes to approve the use of phage-based treatments.^149^ Creating phage banks and personalized phage therapy methods, such as those being developed at Yale University, is essential to designing phage therapies for individual patients and particular bacterial strains. As research progresses, phage therapy promises to revolutionize the treatment of MDR bacterial infections in our time.^150^

## 4 Conclusion

Regulators and stakeholders have raised concerns about the cytotoxicity, immunogenicity, and limited host spectrum of phage therapy. However, the emergence of bacteria that are resistant to drugs has opened a new path for phage-based therapies. Self-replicating and self-limiting phages may be directed toward bacterial receptors critical to pathogenesis. There are diverse phages in nature that appear unharmful to eukaryotic cells. Phages are simple and inexpensive to isolate, propagate, and store. The use of phage in agriculture, unlike chemical pesticide-based methods, can be environmentally beneficial. Furthermore, animal models can be used to assess the safety profile of particular phage before they are administered to humans, which helps expedite the identification of effective phage prior to clinical studies. Using a variety of phages with different host ranges is essential to reduce the possibility of phage-resistant bacterial strains emerging. Phage pre-screening in animal models helps ensure the safety of modified phage. Animal models are therefore essential for studying and eventually implementing phage therapy into human clinical practice.^134^

## Funding

Resources and expenses for this research were performed without external support—all support was entirely from personal or institutional support made by authors and their affiliated institutions. Financial support had no bearing on the integrity or quality of the research used.

## CRediT authorship contribution statement

**Omor Faruk:** Writing – review & editing, Writing – original draft, Visualization, Supervision, Software, Resources, Project administration, Methodology, Investigation, Funding acquisition, Formal analysis, Data curation, Conceptualization. **Zilhas Ahmed Jewel:** Formal analysis, Data curation, Visualization, Resources. **Sanjoy Bairagi:** Formal analysis, Data curation. **Mohammad Rasheduzzaman:** Methodology, Funding acquisition. **Hindol Bagchi:** Formal analysis, Data curation, Visualization, Resources. **Akber Subahan Mahbub Tuha:** Software, Methodology, Funding acquisition. **Imran Hossain:** Supervision, Funding acquisition, Data curation. **Ayon Bala:** Visualization, Resources, Project administration, Methodology. **Sarafat Ali:** Supervision, Methodology, Investigation.

## References

[1] Kasman L.M., Porter L.D. (2023). Bacteriophages. Brenner’s Encycl. Genet.

[2] Klumpp J., Dunne M., Loessner M.J. (2023). A perfect fit: bacteriophage receptor-binding proteins for diagnostic and therapeutic applications. Curr Opin Microbiol.

[3] Sulakvelidze A., Alavidze Z., Morris J.G. (2001). Bacteriophage therapy. Antimicrob Agents Chemother.

[4] Venturini C., Petrovic Fabijan A., Fajardo Lubian A. (2022). Biological foundations of successful bacteriophage therapy. EMBO Mol Med.

[5] García-Cruz J.C., Huelgas-Méndez D., Jiménez-Zúñiga JS (2023). Myriad applications of bacteriophages beyond phage therapy. PeerJ.

[6] Uchechukwu C.F., Shonekan A. (2024). Current status of clinical trials for phage therapy. J Med Microbiol.

[7] Walter N., Mirzaei M., Deng L. (2024). The potential of bacteriophage therapy as an alternative treatment approach for antibiotic-resistant infections. Med Princ Pract.

[8] Han P.J., Lin W., Fan H.H. (2024). Characterization of phage evolution and phage resistance in drug-resistant *Stenotrophomonas maltophilia*. J Virol.

[9] Chung K.M., Liau X.L., Tang S.S. (2023). Bacteriophages and their host range in multidrug-resistant bacterial disease treatment. Pharmaceuticals.

[10] Yang Q., Le S., Zhu T., Wu N. (2023). Regulations of phage therapy across the world. Front Microbiol.

[11] Suh G.A., Lodise T.P., Tamma P.D. (2022). Considerations for the use of phage therapy in clinical practice. Antimicrob Agents Chemother.

[12] Cui L.Z., Kiga K., Kondabagil K. (2024). Current and future directions in bacteriophage research for developing therapeutic innovations. Sci Rep.

[13] Shymialevich D., Wójcicki M., Wardaszka A. (2022). Application of lytic bacteriophages and their enzymes to reduce saprophytic bacteria isolated from minimally processed plant-based food products-In vitro studies. Viruses.

[14] Campbell A. (1961). Conditions for the existence of bacteriophage. Evolution (N Y).

[15] Stone E., Campbell K., Grant I. (2019). Understanding and exploiting phage-host interactions. Viruses.

[16] Grabowski Ł., Łepek K., Stasiłojć M. (2021). Bacteriophage-encoded enzymes destroying bacterial cell membranes and walls, and their potential use as antimicrobial agents. Microbiol Res.

[17] Rogovski P., Cadamuro R.D., da Silva R. (2021). Uses of bacteriophages as bacterial control tools and environmental safety indicators. Front Microbiol.

[18] Gao Z., Feng Y. (2023). Bacteriophage strategies for overcoming host antiviral immunity. Front Microbiol.

[19] Aslam B., Wang W., Arshad M.I. (2018). Antibiotic resistance: a rundown of a global crisis. Infect Drug Resist.

[20] Martínez J.L., Baquero F. (2014). Emergence and spread of antibiotic resistance: setting a parameter space. Ups J Med Sci.

[21] Palmer GH, Buckley GJ, National Academies of Sciences (2021). Combating Antimicrobial Resistance and Protecting the Miracle of Modern Medicine.

[22] Tao S., Chen H., Li N. (2022). The spread of antibiotic resistance genes *in vivo* model. Can J Infect Dis Med Microbiol.

[23] Coleman J.P., Smith C.J. (2014). Microbial resistance. Ref Modul Biomed Sci.

[24] Eliopoulos G.M., Cosgrove S.E., Carmeli Y. (2003). The impact of antimicrobial resistance on health and economic outcomes. Clin Infect Dis.

[25] Lin D.M., Koskella B., Lin H.C. (2017). Phage therapy: an alternative to antibiotics in the age of multi-drug resistance. World J Gastrointest Pharmacol Ther.

[26] Taati Moghadam M., Amirmozafari N., Shariati A. (2020). How phages overcome the challenges of drug resistant bacteria in clinical infections. Infect Drug Resist.

[27] Au A., Lee H., Ye T. (2021). Bacteriophages: combating antimicrobial resistance in food-borne bacteria prevalent in agriculture. Microorganisms.

[28] Noor I., Nasir M.H., Ur Rehman A. (2024). Medicinal and immunological aspects of bacteriophage therapy to combat antibiotic resistance. Explor Med.

[29] Maimaiti Z., Li Z., Xu C. (2023). Research progress of phage therapy in orthopedic implant-related infection. Chin J Surg.

[30] Mishra V., Bankar N., Tiwade Y. (2024). How phage therapy works, its advantages and disadvantages: mini review. J Pure Appl Microbiol.

[31] Zalewska-Piątek B. (2023). Phage therapy—Challenges, opportunities and future prospects. Pharmaceuticals.

[32] Cui L., Watanabe S., Miyanaga K. (2024). A comprehensive review on phage therapy and phage-based drug development. *Antibiotics: Basel*.

[33] Jo S.J., Kwon J., Kim S.G. (2023). The biotechnological application of bacteriophages: what to do and where to go in the middle of the post-antibiotic era. Microorganisms.

[34] Abd-El Wahab A., Basiouni S., El-Seedi H.R. (2023). An overview of the use of bacteriophages in the poultry industry: successes, challenges, and possibilities for overcoming breakdowns. Front Microbiol.

[35] Abdelkader K., Gerstmans H., Saafan A. (2019). The preclinical and clinical progress of bacteriophages and their lytic enzymes: the parts are easier than the whole. Viruses.

[36] Bhargava K., Nath G., Bhargava A. (2021). Phage therapeutics: from promises to practices and prospectives. Appl Microbiol Biotechnol.

[37] Divya Ganeshan S., Hosseinidoust Z (2019). Phage therapy with a focus on the human microbiota. *Antibiotics: Basel*.

[38] Łobocka M., Dąbrowska K., Górski A. (2021). Engineered bacteriophage therapeutics: rationale, challenges and future. BioDrugs.

[39] Salmond G.P.C., Fineran P.C. (2015). A century of the phage: past, present and future. Nat Rev Microbiol.

[40] Kovacs C.J., Rapp E.M. (2024). Disruption of biofilm by bacteriophages in clinically relevant settings. Mil Med.

[41] Pallavali R.R., Degati V.L., Lomada D. (2017). Isolation and *in vitro* evaluation of bacteriophages against MDR-bacterial isolates from septic wound infections. PLoS One.

[42] Chen X.F., Hou X., Xiao M. (2021). Matrix-assisted laser desorption/ionization time of flight mass spectrometry (MALDI-TOF MS) analysis for the identification of pathogenic microorganisms: a review. Microorganisms.

[43] Brown T.L., Petrovski S., Chan H.T. (2018). Semi-solid and solid dosage forms for the delivery of phage therapy to epithelia. Pharmaceuticals.

[44] Colom J., Cano-Sarabia M., Otero J. (2017). Microencapsulation with alginate/CaCO_3_: a strategy for improved phage therapy. Sci Rep.

[45] Liu S.Y., Lu H.Y., Zhang S.L. (2022). Phages against pathogenic bacterial biofilms and biofilm-based infections: a review. Pharmaceutics.

[46] Hosseininasab S.S., Naderifar M., Akbarizadeh M.R. (2024). The use of phages for the biosynthesis of metal nanoparticles and their biological applications: a review. J Cell Mol Med.

[47] El Haddad L., Harb C.P., Gebara M.A. (2019). A systematic and critical review of bacteriophage therapy against multidrug-resistant ESKAPE organisms in humans. Clin Infect Dis.

[48] Schooley R.T., Biswas B., Gill J.J. (2017). Development and use of personalized bacteriophage-based therapeutic cocktails to treat a patient with a disseminated resistant *Acinetobacter baumannii* infection. Antimicrob Agents Chemother.

[49] Gill J.J., Hyman P. (2010). Phage choice, isolation, and preparation for phage therapy. Curr Pharm Biotechnol.

[50] Keen E.C. (2012). Phage therapy: concept to cure. Front Microbiol.

[51] Abedon S.T., Kuhl S.J., Blasdel B.G. (2011). Phage treatment of human infections. Bacteriophage.

[52] Beaber J.W., Hochhut B., Waldor M.K. (2004). *SOS* response promotes horizontal dissemination of antibiotic resistance genes. Nature.

[53] Kohanski M.A., Dwyer D.J., Collins J.J. (2010). How antibiotics kill bacteria: from targets to networks. Nat Rev Microbiol.

[54] Cui H.Y., Yuan L., Lin L. (2017). Novel chitosan film embedded with liposome-encapsulated phage for biocontrol of *Escherichia coli* O157: H7 in beef. Carbohydr Polym.

[55] Mahichi F., Synnott A.J., Yamamichi K. (2009). Site-specific recombination of T2 phage using IP008 long tail fiber genes provides a targeted method for expanding host range while retaining lytic activity. FEMS Microbiol Lett.

[56] Ranveer S.A., Dasriya V., Ahmad M.F. (2024). Positive and negative aspects of bacteriophages and their immense role in the food chain. NPJ Sci Food.

[57] Maura D., Galtier M., Le Bouguénec C. (2012). Virulent bacteriophages can target O104: H4 enteroaggregative *Escherichia coli* in the mouse intestine. Antimicrob Agents Chemother.

[58] Mattila S., Ruotsalainen P., Jalasvuori M. (2015). On-demand isolation of bacteriophages against drug-resistant bacteria for personalized phage therapy. Front Microbiol.

[59] Oechslin F. (2018). Resistance development to bacteriophages occurring during bacteriophage therapy. Viruses.

[60] Hosseinidoust Z., Tufenkji N., van de Ven T.G.M. (2013). Formation of biofilms under phage predation: considerations concerning a biofilm increase. Biofouling.

[61] Jassim S.A.A., Limoges R.G. (2014). Natural solution to antibiotic resistance: bacteriophages ‘the living drugs. World J Microbiol Biotechnol.

[62] Ho M.K.Y., Zhang P., Chen X. (2022). Bacteriophage endolysins against gram-positive bacteria, an overview on the clinical development and recent advances on the delivery and formulation strategies. Crit Rev Microbiol.

[63] Zhang Q.G., Buckling A. (2012). Phages limit the evolution of bacterial antibiotic resistance in experimental microcosms. Evol Appl.

[64] Elfadadny A., Ragab R.F., AlHarbi M. (2024). Antimicrobial resistance of *Pseudomonas aeruginosa*: navigating clinical impacts, current resistance trends, and innovations in breaking therapies. Front Microbiol.

[65] Roy S., Mukherjee P., Kundu S. (2024). Microbial infections in burn patients. Acute Crit Care.

[66] Norbury W., Herndon D.N., Tanksley J. (2016). Infection in burns. *Surg Infect: Larchmt*.

[67] Rios A.C., Moutinho C.G., Pinto F.C. (2016). Alternatives to overcoming bacterial resistances: state-of-the-art. Microbiol Res.

[68] McVay C.S., Velásquez M., Fralick J.A. (2007). Phage therapy of *Pseudomonas aeruginosa* infection in a mouse burn wound model. Antimicrob Agents Chemother.

[69] Durr H.A., Leipzig N.D. (2023). Advancements in bacteriophage therapies and delivery for bacterial infection. Mater Adv.

[70] Lopes-Pacheco M. (2016). CFTR modulators: shedding light on precision medicine for cystic fibrosis. Front Pharmacol.

[71] Veit G., Avramescu R.G., Chiang A.N. (2016). From CFTR biology toward combinatorial pharmacotherapy: expanded classification of cystic fibrosis mutations. Mol Biol Cell.

[72] Rutter W.C., Burgess D.R., Burgess D.S. (2017). Increasing incidence of multidrug resistance among cystic fibrosis respiratory bacterial isolates. Microb Drug Resist.

[73] Bernut A., Dupont C., Ogryzko N.V. (2019). CFTR protects against *Mycobacterium abscessus* infection by fine-tuning host oxidative defenses. Cell Rep.

[74] Dedrick R.M., Guerrero-Bustamante C.A., Garlena R.A. (2019). Engineered bacteriophages for treatment of a patient with a disseminated drug-resistant *Mycobacterium abscessus*. Nat Med.

[75] Law N., Logan C., Yung G. (2019). Successful adjunctive use of bacteriophage therapy for treatment of multidrug-resistant *Pseudomonas aeruginosa* infection in a cystic fibrosis patient. Infection.

[76] Ng R.N., Tai A.S., Chang B.J. (2021). Overcoming challenges to make bacteriophage therapy standard clinical treatment practice for cystic fibrosis. Front Microbiol.

[77] Subramanian A. (2024). Emerging roles of bacteriophage-based therapeutics in combating antibiotic resistance. Front Microbiol.

[78] Frampton R.A., Pitman A.R., Fineran P.C. (2012). Advances in bacteriophage-mediated control of plant pathogens. Int J Microbiol.

[79] Vila M.M.D.C., Balcão L.M.N., Balcão V.M. (2024). Phage delivery strategies for biocontrolling human, animal, and plant bacterial infections: state of the art. Pharmaceutics.

[80] Siyanbola K.F., Ejiohuo O. (2024). Bacteriophages: sustainable and effective solution for climate-resilient agriculture. Sustain Microbiol.

[81] Fortuna K.J., Szoboszlay M., Holtappels D. (2023). Assessing the environmental biosafety of phage-based biocontrol applications. Biol Contr.

[82] Fujiki J., Schnabl B. (2023). Phage therapy: targeting intestinal bacterial microbiota for the treatment of liver diseases. JHEP Rep.

[83] Balcha F.B., Neja S.A. (2023). CRISPR-Cas9 mediated phage therapy as an alternative to antibiotics. Anim Dis.

[84] Emencheta S.C., Onugwu A.L., Kalu C.F. (2024). Bacteriophages as nanocarriers for targeted drug delivery and enhanced therapeutic effects. Mater Adv.

[85] Svircev A., Roach D., Castle A. (2018). Framing the future with bacteriophages in agriculture. Viruses.

[86] Ferriol-González C., Domingo-Calap P. (2021). Phage therapy in livestock and companion animals. Antibiotics.

[87] McFall-Ngai M., Hadfield M.G., Bosch T.C. (2013). Animals in a bacterial world, a new imperative for the life sciences. Proc Natl Acad Sci USA.

[88] Penziner S., Schooley R.T., Pride D.T. (2021). Animal models of phage therapy. Front Microbiol.

[89] Nabil N.M., Tawakol M.M., Samir A. (2024). Evaluation of lyophilized bacteriophage cocktail efficiency against multidrug-resistant *Salmonella* in broiler chickens. BMC Microbiol.

[90] Sarrami Z., Sedghi M., Mohammadi I. (2023). Effects of bacteriophage on *Salmonella enteritidis* infection in broilers. Sci Rep.

[91] Manohar P., Ramesh N (2019). Improved lyophilization conditions for long-term storage of bacteriophages. Sci Rep.

[92] Kowalska J.D., Kazimierczak J., Sowińska P.M. (2020). Growing trend of fighting infections in aquaculture environment-opportunities and challenges of phage therapy. Antibiotics.

[93] Muliya Sankappa N., Shivani Kallappa G., Kallihosuru Boregowda K. (2024). Novel lytic bacteriophage AhFM11 as an effective therapy against hypervirulent Aeromonas hydrophila. Sci Rep.

[94] Dong J., Zhang D.F., Li J.R. (2021). Genistein inhibits the pathogenesis of *Aeromonas hydrophila* by disrupting quorum sensing mediated biofilm formation and aerolysin production. Front Pharmacol.

[95] Kutter E., Sulakvelidze A., Bacteriophages: Biology and Applications - Google Books. CRC Press, 2004. M12 28 - 528. https://books.google.com.bd/books?hl=en&lr=&id=6sTBd2EJpmYC&oi=fnd&pg=PA91&dq=Sulakvelidze+et+al.,+2001a)+phage&ots=j29P0dk-jw&sig=LrJnI81f_WVethM7Iv2VtocRoVw&redir_esc=y#v=onepage&q=Sulakvelidzeetal.%2C2001a)phage&f=false (Accessed November 02, 2024).

[96] Colavecchio A., Cadieux B., Lo A. (2017). Bacteriophages contribute to the spread of antibiotic resistance genes among foodborne pathogens of the *Enterobacteriaceae* family - A review. Front Microbiol.

[97] Torres-Barceló C. (2018). The disparate effects of bacteriophages on antibiotic-resistant bacteria. Emerg Microbes Infect.

[98] Kęsik-Szeloch A., Drulis-Kawa Z., Weber-Dąbrowska B. (2013). Characterising the biology of novel lytic bacteriophages infecting multidrug resistant *Klebsiella pneumoniae*. Virol J.

[99] Liu D., Van Belleghem J.D., de Vries C.R. (2021). The safety and toxicity of phage therapy: a review of animal and clinical studies. Viruses.

[100] Murtazalieva K., Mu A., Petrovskaya A. (2024). The growing repertoire of phage anti-defence systems. Trends Microbiol.

[101] Parracho H.M., Burrowes B.H., Enright M.C. (2012). The role of regulated clinical trials in the development of bacteriophage therapeutics. J Mol Genet Med.

[102] Liu S., Quek S.Y., Huang K. (2024). Advanced strategies to overcome the challenges of bacteriophage-based antimicrobial treatments in food and agricultural systems. Crit Rev Food Sci Nutr.

[103] Andersson D.I. (2006). The biological cost of mutational antibiotic resistance: any practical conclusions?. Curr Opin Microbiol.

[104] Würstle S., Lee A., Kortright K.E. (2024). Optimized preparation pipeline for emergency phage therapy against *Pseudomonas aeruginosa* at Yale University. Sci Rep.

[105] Caflisch K.M., Suh G.A., Patel R. (2019). Biological challenges of phage therapy and proposed solutions: a literature review. Expert Rev Anti Infect Ther.

[106] Sithu Shein A.M., Hongsing P., Khatib A. (2024). Phage therapy could be key to conquering persistent bacterial lung infections in children. NPJ Antimicrob Resist.

[107] Hyman P. (2019). Phages for phage therapy: isolation, characterization, and host range breadth. Pharmaceuticals.

[108] Pirnay J.P., Djebara S., Steurs G. (2024). Personalized bacteriophage therapy outcomes for 100 consecutive cases: a multicentre, multinational, retrospective observational study. Nat Microbiol.

[109] Mahmud M.R., Tamanna S.K., Akter S. (2024). Role of bacteriophages in shaping gut microbial community. Gut Microbes.

[110] Yu X., Cheng L., Yi X. (2024). Gut phageome: challenges in research and impact on human microbiota. Front Microbiol.

[111] Yoo S., Lee K.M., Kim N. (2024). Designing phage cocktails to combat the emergence of bacteriophage-resistant mutants in multidrug-resistant *Klebsiella pneumoniae*. Microbiol Spectr.

[112] Vaitekenas A., Tai A.S., Ramsay J.P. (2021). *Pseudomonas aeruginosa* resistance to bacteriophages and its prevention by strategic therapeutic cocktail formulation. Antibiotics.

[113] Chan B.K., Abedon ST (2012). Phage therapy pharmacology phage cocktails. Adv Appl Microbiol.

[114] Goodridge L. (2010). Designing phage therapeutics. Curr Pharm Biotechnol.

[115] Meile S., Du J.M., Dunne M. (2022). Engineering therapeutic phages for enhanced antibacterial efficacy. Curr Opin Virol.

[116] Koskella B., Meaden S. (2013). Understanding bacteriophage specificity in natural microbial communities. Viruses.

[117] Marinelli L.J., Piuri M., Swigonová Z. (2008). BRED: a simple and powerful tool for constructing mutant and recombinant bacteriophage genomes. PLoS One.

[118] Lv S., Wang Y., Jiang K. (2023). Genetic engineering and biosynthesis technology: keys to unlocking the chains of phage therapy. Viruses.

[119] Scholl D., Adhya S., Merril C (2005). *Escherichia coli* K1’s capsule is a barrier to bacteriophage T7. Appl Environ Microbiol.

[120] Yoichi M., Abe M., Miyanaga K. (2005). Alteration of tail fiber protein gp38 enables T2 phage to infect *Escherichia coli* O157: H7. J Biotechnol.

[121] Łoś J., Zielińska S., Krajewska A., Harper D.R., Abedon S.T., Burrowes B.H., McConville M.L. (2021). Bacteriophages.

[122] Hibstu Z., Hibstu Z., Belew H. (2022). Phage therapy: a different approach to fight bacterial infections. Biologics.

[123] Gordillo Altamirano F.L., Barr J.J (2019). Phage therapy in the postantibiotic era. Clin Microbiol Rev.

[124] Uyttebroek S., Chen B.X., Onsea J. (2022). Safety and efficacy of phage therapy in difficult-to-treat infections: a systematic review. Lancet Infect Dis.

[125] Grigson S.R., Giles S.K., Edwards R.A. (2023). Knowing and Naming: phage annotation and nomenclature for phage therapy. Clin Infect Dis.

[126] Onallah H., Yerushalmy O., Braunstein R. (2024). Protocol for phage matching, treatment, and monitoring for compassionate bacteriophage use in non-resolving infections. STAR Protoc.

[127] Luong T., Salabarria A.C., Roach D.R. (2020). Phage therapy in the resistance era: where do we stand and where are we going?. Clin Ther.

[128] Łusiak-Szelachowska M., Międzybrodzki R., Drulis-Kawa Z. (2022). Bacteriophages and antibiotic interactions in clinical practice: what we have learned so far. J Biomed Sci.

[129] Berkson J.D., Wate C.E., Allen G.B. (2024). Phage-specific immunity impairs efficacy of bacteriophage targeting vancomycin resistant *Enterococcus* in a murine model. Nat Commun.

[130] Hsu B.B., Gibson T.E., Yeliseyev V. (2019). Dynamic modulation of the gut microbiota and metabolome by bacteriophages in a mouse model. Cell Host Microbe.

[131] Koskella B., Brockhurst M.A. (2014). Bacteria-phage coevolution as a driver of ecological and evolutionary processes in microbial communities. FEMS Microbiol Rev.

[132] Kaprou G.D., Bergšpica I., Alexa E.A. (2021). Rapid methods for antimicrobial resistance diagnostics. Antibiotics.

[133] Jian Z.H., Zeng L., Xu T.J. (2021). Antibiotic resistance genes in bacteria: occurrence, spread, and control. J Basic Microbiol.

[134] Bleriot I., Pacios O., Blasco L. (2024). Improving phage therapy by evasion of phage resistance mechanisms. JAC Antimicrob Resist.

[135] Samananda S.L. (2024). Nano-emulsion encapsulation for the efficient delivery of bacteriophage therapeutics. Biologicals.

[136] Wang B., Du L., Dong B.P. (2024). Current knowledge and perspectives of phage therapy for combating refractory wound infections. Int J Mol Sci.

[137] Malik D.J., Resch G. (2020). Editorial: manufacturing, formulation and delivery issues for phage therapy to become a reality. Front Microbiol.

[138] Rahmani R., Zarrini G., Sheikhzadeh F. (2015). Effective phages as green antimicrobial agents against antibiotic-resistant hospital *Escherichia coli*. Jundishapur J Microbiol.

[139] Chhibber S., Kaur J., Kaur S. (2018). Liposome entrapment of bacteriophages improves wound healing in a diabetic mouse MRSA infection. Front Microbiol.

[140] Wall S.K., Zhang J.Y., Rostagno M.H. (2010). Phage therapy to reduce preprocessing *Salmonella* infections in market-weight swine. Appl Environ Microbiol.

[141] Rombouts S., Volckaert A., Venneman S. (2016). Characterization of novel bacteriophages for biocontrol of bacterial blight in leek caused by *Pseudomonas syringae* pv. porri. Front Microbiol.

[142] Lee C., Kim H., Ryu S. (2023). Bacteriophage and endolysin engineering for biocontrol of food pathogens/pathogens in the food: recent advances and future trends. Crit Rev Food Sci Nutr.

[143] Wittmann J., Brancato C., Berendzen K.W. (2016). Development of a tomato plant resistant to *Clavibacter michiganensis* using the endolysin gene of bacteriophage CMP1 as a transgene. Plant Pathol.

[144] Doss J., Culbertson K., Hahn D. (2017). A review of phage therapy against bacterial pathogens of aquatic and terrestrial organisms. Viruses.

[145] Opperman C.J., Wojno J.M., Brink A.J. (2022). Treating bacterial infections with bacteriophages in the 21st century. S Afr J Infect Dis.

[146] Eghbalpoor F., Gorji M., Alavigeh M.Z. (2024). Genetically engineered phages and engineered phage-derived enzymes to destroy biofilms of antibiotics resistance bacteria. Heliyon.

[147] Li X.H., He Y.H., Wang Z.L., et al. A combination therapy of phages and antibiotics: two is better than one. *Int J Biol Sci*., 17(13): 3573–3582. doi:10.7150/ijbs.60551. 10.7150/ijbs.60551 PMC8416725 34512166

[148] Nale J.Y., Clokie M.R. (2021). Preclinical data and safety assessment of phage therapy in humans. Curr Opin Biotechnol.

[149] Lewis J.M., Williams J., Sagona A.P. (2024). Making the leap from technique to treatment - genetic engineering is paving the way for more efficient phage therapy. Biochem Soc Trans.

[150] Kapoor A., Mudaliar S.B., Bhat V.G. (2024). Phage therapy: a novel approach against multidrug-resistant pathogens. 3 Biotech.

